# Relation between interleukin-6 concentrations and oxidative status of HIV infected patients with /or at risk of Kaposi disease in Yaounde

**DOI:** 10.1186/s12985-023-02109-9

**Published:** 2023-07-25

**Authors:** Roger Ahouga Voufo, Armand Emmanuel Kouotou, Nchinda Jones Tatah, Georges TeTo, Cédric Gueguim, Chantal Marie Essome Ngondé, Armand Gabin Njiguet Tepa, Arnaud Gabin, Falmata Amazia, Natacha Lena Yembeau, Charles Kouanfack, Pieme Constant Anatole

**Affiliations:** 1 Institute of Medical Research and Medicinal Plants Studies (IMPM), Yaounde, Cameroon; 2 Department of Biochemistry, Faculty of Medicine and Biomedical Sciences, University of Yaoundé I, Yaounde, Cameroon; 3 Department of Internal Medicine Faculty of Medicine and Biomedical Sciences, University of Yaoundé I, Yaounde, Cameroon; 4 Centre International de Référence Chantal Biya (CICRB), Yaounde, Cameroon; 5 Department of Biochemistry, Faculty of Science, University of Dschang, Dschang, Cameroon; 6 Faculty of Medicine and pharmaceutical Sciences, University of Dschang, Dschang, Cameroon

**Keywords:** Interleukin-6, Oxidative status, Kaposi’s disease, HIV

## Abstract

**Objective:**

To investigate the relation between interleukin-6 concentration and oxidative status of HIV infected patients with or at risk of Kaposi’s disease in Yaoundé.

**Methods:**

We conducted a two-months cross-sectional study on 87 consenting HIV infected patients followed at the Day Hospital of the Yaoundé Central Hospital. Serum/plasma obtained after centrifugation of blood collected in dry/EDTA tubes was used for the determination of Human Herpes Virus-8 antigen (HHV-8) and Interleukin-6 (IL-6) by the ELISA technique, and that of oxidative stress markers: Malondialdehyde (MDA) reduced Glutathione (GSH) and total antioxidant capacity by spectrophotometry.

**Results:**

Subjects belonging to the [40–50[year-old age group were mainly represented in our study population with 43.7%. The average age was 44.6 ± 10.4 years with extremes ranging from 26 to 72 years. The sex ratio was 0.24. Our population was mainly represented by people infected with HIV type I (90.8%) and 3.4% had developed clinical signs of Kaposi’s disease. The prevalence of the HHV-8 antigen was 57.5%. Immune and oxidative parameters did not vary with age, sex and therapeutic line. We noted a significant increase in IL-6 concentrations in patients positive to the HHV-8 antigen for IL-6 concentrations < 37 (P = 0.005; CI= [0.40; 0.59]. MDA and GSH concentrations increased significantly with the HHV-8 infection (P < 0.0001; CI= [0.40; 0.59] and P < 0.0001; CI= [13.30;21.45], respectively). Total antioxidant capacity (FRAP) decreased significantly with HHV-8 infection (P = 0.004; CI= [-69.18; -13.78]). We noted a significant increase in MDA concentrations in patients taking their ARVs irregularly, (P < 0.0001).

**Conclusion:**

Our study showed a weak positive correlation between IL-6 and MDA, a strong negative correlation between FRAP and MDA and a strong positive correlation between MDA and GSH highlighting the association of these few markers of oxidative stress and Il-6 to the risk of Kaposi’s disease.

## Introduction

Kaposi’s disease (KD) is a multifocal angiogenic process characterized by a double vascular and cellular proliferation, multicentered, the neoplastic nature of which remains argued [1]. It is described in four epidemiological forms [2]. Indeed, the observations showed that the risk of developing KD in HIV patients was 200,000 times higher than in the general population [3]. KD is one of the most common opportunistic diseases and the first cancer in people living with HIV (PLHIV) [4].

The seroprevalence of HHV-8 varies from 2 to 7% in Western Europe and North America, from 10 to 20% in the Mediterranean region and is 100% in some regions of Sub-Saharan Africa [5]. HHV-8 infects monocytes and B lymphocytes and induces in these cells the production of factors that make up the developmental bed of KD lesions [5]. One of the unresolved factors is the event that triggers the process: it is linked not only to HHV-8 but also to external factors such as an inflammatory lesion, cytokinetic disorder or oxidative stress [6].The HHV-8 genome allows it to potentially express genes that can induce fusiform cell development and stimulate angiogenesis. Among these genes, we can note a viral homolog of interleukin 6, homologs of “macrophage inflammatory protein” (MIP) and one homolog of the K3/K5 protein receptor of HHV-8 [7]. In the case of HIV, the increased stimulation of certain cell subpopulations (lymphocytes, phagocytes) and the action of certain infectious agents leads to the production of reactive oxygen species (ROS) [8]. Thus, in an inflammatory focus, stimulated phagocytic cells are a source of ROS production. Free radicals act on the NF-kB transcription factor that promotes HIV replication [9]. ROS, which are cellular intermediates, influence cellular signaling pathways, which are mainly mediated by transcription factors such as NF-kB, cytokines, etc. The accumulation of ROS can cause several disorders including carcinogenesis [10].

Cameroun is an endemic area for HHV-8 [11] and the immune system of Cameroonians would therefore be constantly stressed, resulting in chronic immune activation against this virus [12]. Given that HIV causes chronic inflammation and that stimulated phagocytic cells in the inflammatory foci are a source of ROS production. We proposed as part of this work to investigate the immune (Interleukin-6) and oxidative status of HIV-positive and HIV patients infected with HHV-8 followed in the Day Hospital of the Yaoundé Central Hospital in order to look for markers that may be involved in the triggering of Kaposi.

## Materials and methods

### Type and place of the study

The Day Care Unit of the Yaoundé Central Hospital served as a framework for this study given its geographical location and its ability to receive a large number of People living with HIV (PLHIV) because it represents the largest management unit for these patients. We conducted a two-months cross-sectional analytical study from October 28 to December 27, 2020 on HIV-infected patients followed at the above-mentioned unit.

### Eligibility and recruitment

Given that the epidemic form of Kaposi disease is an opportunistic disease upon HIV infection, all participants of this study were HIV positive. All patients were recruited consecutively at the level of the management unit (MU) of PLHIV at the day hospital. Included in our study were any HIV-infected patient followed at the Day Hospital of the Yaoundé Central Hospital having freely consented to participate in our study. This study did not include any HIV positive Patient undergoing any other therapy than ARV as well as any patient who withdrew consent during the study. Based on these criteria, we obtained 87 participants of both sexes aged between 26 and 72 years.

### Ethical consideration

The study was conducted in patients whose identity and address remained confidential. The target population was informed in advance and we retained only those who had freely gave their consent. Ethical clearances were issued by the Centre Regional Ethics Committee for Human Health Research (CE-1567/CRERSHC/2020), Institutional Ethics Committee (295/UYI/FMSB/VDRC/DAASR/CSD) and Research as well as research authorizations from the Directorate of the Yaoundé Central Hospital (179/20/AR/DHCY/CM/SM) and the Directorate of the Imagery Centre and Biological Analysis EXACT (0001/21/CIAB EXACT/RH/DG) that permitted us to carry out this study as designed.

### Data collection and processing methods

All patients coming to the management unit of PLHIV during our collection period were informed of our study through an information leaflet followed by a proposal for informed consent. Prior to the deduction, those who freely consented to participate were subject to a questionnaire (Marital status, Educational level, Age, Sex…).

### Sample collection and retention

Venous blood was collected from each subject in a dry/EDTA tube while respecting the general rules of asepticism, hygiene and safety in the laboratory. These samples were centrifuged (1500 g for 5 min) and the serum/plasma aliquoted in triplicates and transported in a cooler containing ice accumul ators to the Biochemistry laboratory of the Faculty of Medicine and Biomedical Sciences (FMSB) or frozen at -20 °C until the time of the analyses. These biological analyses were carried out in the immune-serology laboratory of the Imagery Centre and Biological Analysis EXACT and the Biochemistry Laboratory of the Faculty of Medicine and Biomedical Sciences (FMSB) of Yaoundé I University.

### Determination of immunological parameters

#### Determination of HHV-8 antigen (HHV-8 ELISA kit mybiosource Cat.No MBS167838)

It was done according to the principle of the indirect ELISA method wherein anti-HHV-8 antibodies are adsorbed on microwells [13]. Positive and negative samples or controls are added to the wells, the HHV-8 antigens in the sample bind to the antibodies adsorbed in the wells, unfixed antigens are eliminated during washing. An enzyme-coupled detection antibody (HRP) is added to wells and incubated. Excess enzyme is eliminated during a washing step and then added a chromogenic substrate. The reaction is then stopped and the optical densities measured at 450 nm. To express the results, the cutoff value is calculated by averaging the optical densities of the negative control + 0.15 and then any sample OD < cutoff is a negative result and any sample OD > cutoff is a positive result.

#### Determination of IL-6 according to the principle of the sandwich ELISA method (IL-6 ELISA Kit Elabscience^R^ Cat.No E-EL-h0102)

In this technique, a specific anti-IL-6 antibody is adsorbed on microwells which binds to the IL-6 present in the sample or standard [14]. A monoclonal anti-IL-6 antibody conjugated to biotin is then added that binds to IL-6 captured by the first antibody. After incubation, the unbound conjugate is removed during a washing step. Streptavidin-HRP is added and binds to the anti-IL-6 biotin conjugate. After incubation, the unfixed streptavidin-HRP is removed during a washing step, and the HRP-reactive substrate solution is added to the wells. A colored product is formed in proportion to the amount of IL-6 present in the sample. The reaction is stopped by adding the stop solution and absorbance is measured at 450 nm. A standard curve is prepared from seven IL-6 standard dilutions and the concentration of the IL-6 sample is obtained from the equation of this curve. In order to have the homogeneous data, we divided the population into two groups with 37 as the threshold.

### Determination of oxidative stress parameters

#### Assessment of serum lipids oxidation

The determination of malondialdehyde (MDA) was done by the Wilbur et al. method [15]. Carbonyl compounds such as malondialdehyde resulting from the decomposition of fatty acid hydroperoxides react with thiobarbituric acid (TBA) to give pink chromophores whose concentration is determined by reading absorbance at 532 nm.

### Determination of serum total antioxidant capacity

The principle is based on the FRAP method (Ferric Reducing Antioxidant Power) of Benzie et al. [16]. This method measures the ability of plasma to reduce ferric iron (Fe3+) to ferrous iron (Fe2+) in an acid environment pH (3.6). The intensity of the blue color formed by ferrous tripyridyltriazine and measured at 593 nm is proportional to the antioxidant power of the sample.

### Determination of reduced glutathione (GSH)

The principle of this method is based on 2,2-dithio-5,5’-dibenzoic acid (DTNB) reacting with the SH groups of glutathione in the samples and standard forming a yellow colored complex that absorbs light at a wavelength of 412 nm [17].

### Determination of HIV Viral load

HIV Viral load was determined using ABI PRISM 7500 Fast Real Time PCR (Applied Biosystems) with a threshold of 40 Copies/mL.

### Data analysis

All data in this study was analyzed using the SPSS 12.0 software. This consisted of expressing the results as mean ± standard error. All comparisons were done using the student t-test and ANOVA. On the other hand, correlations between Kaposi’s disease, immunological and biochemical parameters were done using the Pearson test. The threshold of significance was set at 5%.

## Results

### Socio-demographic characteristics

The distribution of our population according to age groups shows that the [40–50[ year-olds range was the most represented with 43.7% percentage. The majority of our participants (80.5%) were female, with a sex ratio of 0.24. Single people were the most represented (42.5%). Our participants were mostly from informal sector workers (87.4%); the highest level of education was secondary school (51.7%) and 59.8% of our population resides in urban areas (Table 1).

**Table 1:** Socio-demographic characteristics

Parameters		Frequency (%)
**Age ranges **(year)	[20–30[	9,2
	[30–40[	18,4
	[40–50[	43,7
	[50–60[	19,5
	[60	9,2
**Gender**	Female	80,5
	Male	19,5
**Matrimonial status**	Divorced	33,3
	Widows	42,5
	Single	18,4
	Married	5,7
**Profession**	Student	2,3
	Formal	10,3
	Informal	87,4
**Level of studies**	Primary	31,0
	Secondary	51,7
	University	17,2
**Residence**	Urban Peri	27,6
	Urban	59,8
	Rural	12,6

### Clinical and therapeutic characteristics

The distribution of the population according to duration of ARV treatment shows that 36.8% of our participants had been on ARV treatment for 5 to 10 years, while those over 15 years duration accounted for 10.3%. Our population was mainly represented by people infected with HIV type I (90.8%) who were all on first-line treatment (90.8%). Clinical signs presented were mainly skin damages including macules, papules and nodules, Clinical and therapeutic frequencies are shown in Table 2.

**Table 2:** Clinical and therapeutic characteristics

Parameters		Frequency/(%)
**Duration of treatment** (year)	[0–5[	31
	[5–10[	36,8
	[10–15[	21,8
	[50–60[	19,5
	[15	10,3
**Type of HIV**	HIV 1	90,8
	HIV 2	8,1
	HIV 1 and 2	1,1
**Therapeutic line**	1st line	90,8
	2nd line	8,1
	3rd line	1,1
**Clinical signs of the Kaposi disease**	No	96,6
	Yes	3,4

### Biological characteristics

#### Population distribution with respect to HHV-8 infection and HIV viral load

Of the 87 participants recorded in this study, 57.5% (50) tested positive for HHV-8 antigen compared to 42.5% (37) negative.

Patients with an undetectable viral load (< 40 copies /mL) accounted for 72.4% (63) of our population compared to 27.6% (24) of patients with a viral load ≥ 40 copies /mL.

**Fig. 1:**
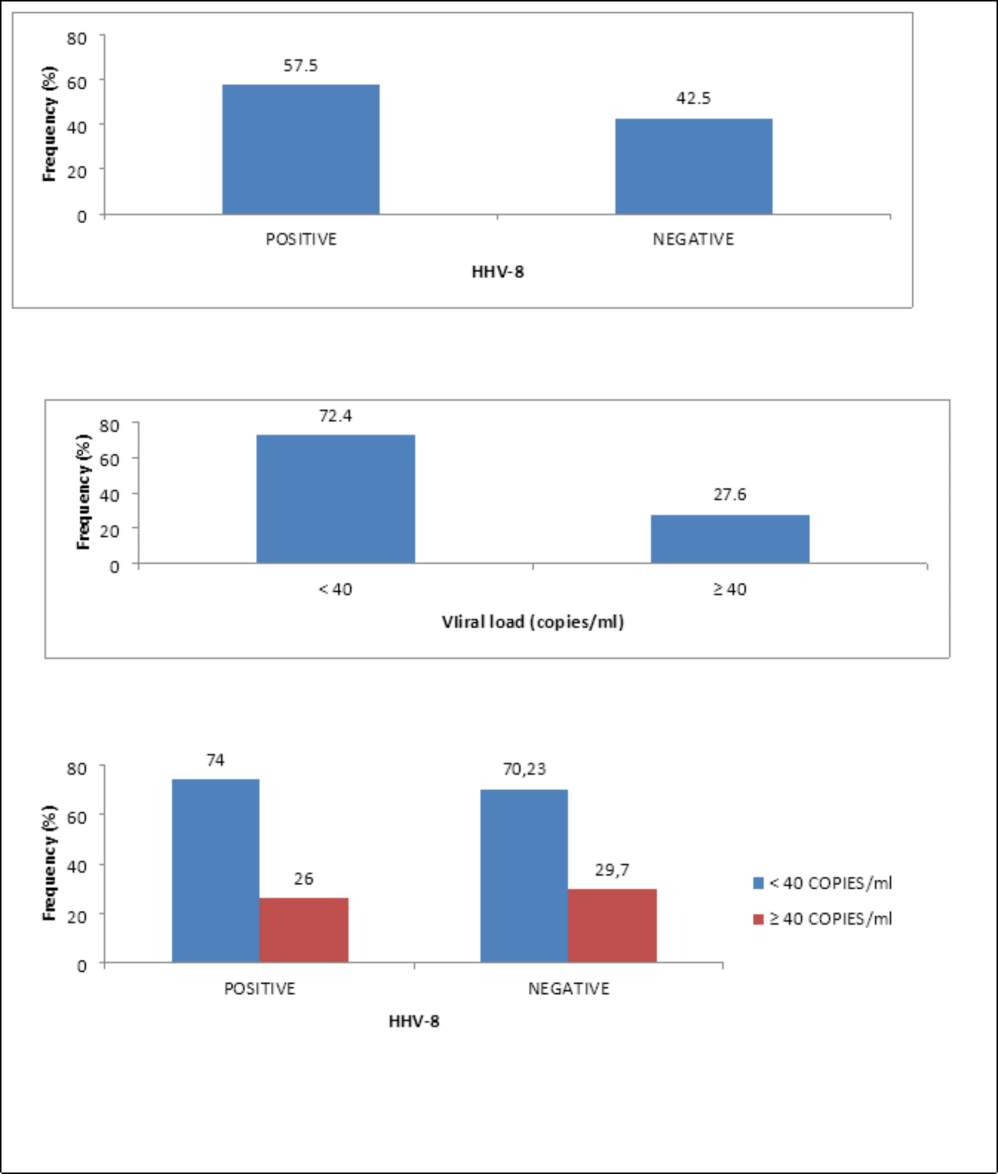
HHV-8 infection and HIV viral load

The variation in viral load with respect to the HHV-8 infection shows that 74% of people positive to HHV-8 infection has a viral load < 40 copies /ml compared to 26% of viral load ≥ 40 copies/ml whereas 70.23 of people negative to HHV-8 infection showed an undetectable viral load (p = 0.8). These informations are shown in Fig. 1.

### Interleukin-6 and oxidative parameters

The IL-6 concentrations of our participants were dispersed with extremes values ranging from 1.2 to 208.9 pg/mL. We had an IL-6 group ≥ 37 pg/ml and the other one < 37 pg/ml in 81 participants (the results obtained from 6 patients were non-exploitable as well as could not be clearly interpreted). Oxidative parameters were measured in 87 participants with the following averages: 1.5 µmol/L; 308.1 mM and 49.7 µmol/L for MDA, FRAP and GSH, respectively.

**Variation of Il-6 concentration and oxidative parameters as a function of the risk of Kaposi’s disease**.

The variation in IL-6 with respect to the HHV-8 infection shows a significant increase in IL-6 concentrations in HHV-8-positive patients for the IL-6 level < 37 pg/mL (*p* < 0.005; CI= [0.40; 0.59] and a non-significant difference in IL-6 level ≥ 37 *p* = 0.84. HHV-8 infection significantly increased MDA and GSH concentrations (*p* < 0.0001; CI= [0.40; 0.59] and *p* < 0.0001; CI = [13.30;21.45] respectively) but significantly decreased Total antioxidant capacity concentrations (FRAP) (*p* = 0.004; CI= [-69.18; -13.78]) (Table 3).

**Table 3:** Variation of immune and oxidative parameters with respect to HHV-8 infection

	HHV-8	N	Mean	*p*	CI
	Positive	15	97,2 ± 53,5		
**IL- 6 ≥ 37(pg/mL)**				0,84	[0,40 ; 0,59]
	Negative	13	93,2 ± 48,2		
	Positive	32	10,2 ± 7,5		
**IL- 6 < 37(pg/mL)**				0,005	[0,40 ; 0,59]
	Negative	21	5,5 ± 3,9		
	Positive	50	1,7 ± 0,2		
**MDA (µmol/L)**				< 0,0001	[0,40 ; 0,59]
	Negative	37	1,2 ± 0,2		
	Positive	50	290,5 ± 39,9		
**FRAP (mM)**				0,004	[-69,18 ; -13,78]
	Negative	37	331,9 ± 76,5		
	Positive	50	57,1 ± 11,4		
**GSH (µmol/L)**				< 0,0001	[13, 30 ; 21, 45]
	Negative	37	39,7 ± 7,6		

*MDA = Malondialdehyde; GSH = Reduced glutathione; FRAP = Total antioxidant capacity.

### Variation of stress parameters with respect to the frequency of ARV intake

Table 4 shows a significant increase in MDA concentrations in patients taking their ARVs irregularly, (*p* < 0.0001). On the other hand, for GSH and total antioxidant capacity, there is no significance.

**Table 4:** Variation of Oxidative stress parameters with respect to ARV intake

	ARV	N	Mean	*P*
**MDA (µmol/L)**	Regular	75	1,5 ± 0,3	< 0,0001
	Irregular	12	1,7 ± 0,1	
**FRAP (mM)**	Regular	75	309,6 ± 64,5	0,43
	Irregular	12	298,8 ± 38,8	
**GSH (µmol/L)**	Regular	75	49,4 ± 13,3	0,52
	Irregular	12	52,0 ± 12,7	

*MDA = Malondialdehyde; GSH = Reduced glutathione; FRAP = Total antioxidant capacity.

### Correlation between Il-6 and oxidative parameters

The association between Il-6 and oxidative parameters shows a weak positive correlation between IL-6 and MDA, a strong negative correlation between FRAP and MDA and a strong positive correlation between MDA and GSH (Table 5).

**Table 5:** Pearson correlation between immunological and oxidative parameters

		IL- 6 (pg/mL)	MDA (µmol/L)	FRAP (mM)	GSH (µmol/L)
**IL- 6 (pg/mL)**	R		0,3^*^		
**MDA (µmol/L)**	R	0,3^*^		-0,5^**^	
					0,4^**^
**FRAP (mM)**	R		-0,5^**^		
**GSH (µmol/L)**	R		0,4^**^		

*. The correlation is significant at the 0.05 level ; **. The correlation is significant at level 0.01.

R = Correlation coefficient ;

## Discussion

Kaposi’s disease is a vascular neoplasia related to Human Herpes Virus-8 infection. The subjects involved in this study were all HIV positive followed at the Day Care Unit of Yaounde Central Hospital. In our population, subjects in the [40–50[years-old range were the most represented (43.7%), with mean age of 44.9 ± 10.4. The sex ratio was 0.24, lower compared to that obtained by Ndouma (2014) in their study, who registered an average age of 37.5 ± 8 years and a sex ratio of 2.0. Similarly, Njiki et al., (2015) reported a sex ratio of 1.6 in their study, with a majority of patients found between 31 and 50 years (60.6%) [11]. A lower sex ratio in our study could be explained by the fact that we used an HIV-infected population without any information on their Kaposi’s disease status meanwhile Ndouma and Njiki in their studies registered a higher sex ratio possibly due to the fact that their studies focused on patients already known positive with Kaposi’s disease given that this pathology mainly attacks male subjects [5]. The population distribution according to the duration of ARVs treatment shows that most of our participants had been on ARV treatment for 5 to 10 years (36.8%). Our population was mainly represented by people infected with HIV type I, 90.8% of who were all subjected to first-line treatment. Indeed, HIV-1 is responsible for almost all AIDS cases worldwide, so a study conducted at the “Centre Pasteur” of Cameroon in 2004 by Jembia et al. found 100% HIV 1 [18].All subjects in this study were on ARV treatment of different lines; first-line treatment [Zidovudine (AZT, ZDV) + Lamivudine (3TC) + Nevirapine (NFV) or Zidovudine (AZT, ZDV) + Lamivudine (3TC) + Efavirenz (EFV) ] was the most administered 90.8%; indeed, any HIV-positive patient eligible for treatment is initially subjected to the first line treatment.

Of the 87 participants recorded in this study, 57.5% (50) were tested positive for the HHV-8 antigen. This result is close to that reported by Mbondji and collaborators in 2013 in Cameroon who found a prevalence of 61% for HHV-8 among PLHIV [19]; and different from those of Njiki and collaborators in 2015 at Yaounde General Hospital who reported a prevalence of 90%. This difference in result would be due to the fact that their study was conducted in the oncology department where the majority of patients showed signs of Kaposi’s disease and also to the fact that they looked at antibodies in their study [12]. we thought that a higher HIV viral load would expose the patient more to HHV-8 infection. Patients with an undetectable viral load (< 40 copies /mL) accounted for 72.4%(63) of our population compared to 27.6%(34) of high viral load (≥ 40 copies /mL). Viral load level is not affected by patients’ HHV-8 status (*p = 0.80*) [20] though effective HIV treatment can reduce the viral load to an “undetectable” level, which significantly reduces the risk of HIV transmission and keeps the patient in apparent health despite the presence of certain pathogens [21].For IL-6 concentrations ≥ 37 pg/mL, the average value was 95.4 ± 50.2 pg/mL with extreme values ranging from 37.1 to 208.9 pg/mL. In addition, for IL-6 concentrations < 37 pg/mL, the average value was 8.3 ± 6.8 pg/mL with extreme values ranging from 1.2 to 24.1 pg/mL. An imbalance in the cytokinetic network is an integral part of the immunopathology of HIV infection. Chronic hyperactivity of the immune system results in spontaneous hyperproduction of pro-inflammatory cytokines: IL6, TNF alpha, IL1, and promotes the replication and spread of the virus in the body. HIV produces inflammations which favours an increase in IL6 levels [22].

In our population, 3.4% showed clinical signs for Kaposi’s disease. Indeed, Tounouga et al., led a cross-sectional cohort in the Day Care Unit of the Yaounde Central Hospital from 2001 to 2016 and found an incidence rate of 2.22% [6]. These results are close to those of Alain GUIMA et al., who reported a prevalence of Kaposi’s disease of 2.54% in a study conducted in Yaounde in 2014 [5]. This prevalence of Kaposi’s disease could be maintained by the discontinued use of ARVs among our participants.

The vascular endothelium is dynamic and very sensitive to oxidative stress. With age, the gradual change from the redox environment to a pro-oxidative state contributes to a gradual redistribution of endothelial factors leading to endothelial dysfunction. It has been amply described that with age, oxidative stress induces molecular damage to lipids, proteins and nucleic acids in different tissues of various species. The mechanisms for repairing this damage and maintaining homeostasis have been identified and are continuously activated throughout life and allow temporary adaptation to multiple aggressions [23]. Evaluation of the variation in immune and oxidative parameters with sex showed no significant difference (*p > 0.05*) [24]. The variation in IL-6 with respect to HHV-8 shows a significant increase in IL-6 concentrations in HHV-8-positive patients for the IL-6 level < 37 pg/mL (*p < 0.01*; CI= [0.40; 0.59] and a non-significant difference in IL-6 level ≥ 37 pg/mL. This might be explained by the fact that the immune response during KD within a sample result in a variation in IL-6 and IL-10 cytokine levels and the presence of HHV-8 [11]. In addition, HHV-8 has a dozen genes, encoding for proteins whose role is to modulate the immune response and regulate cell proliferation and differentiation. These genes would have been integrated by HHV-8 during its evolution, HHV-8 has a gene that encodes for a protein close to human interleukin 6 (vIL-6), whose role seems to be pivotal in the pathogenesis of Kaposi’s disease. An evaluation of the levels of several proteins associated with inflammation: IL-6, D-dimer and C-reactive protein enabled the association of high levels of IL-6 with a 40% increase in cancer risk [25].

The concentrations of MDA and GSH increased significantly with HHV-8 infection (*p < 0.0001*; CI= [0.40; 0.59] and *p < 0.0001*; CI= [13.30;21.45] respectively). HHV-8 has been reported to cause molecular damage due to the activity of the HIV-1 transcription Transactivator (Tat) protein, which influences the cellular redox state by two mechanisms; by decreasing antioxidant concentrations including the collapse of manganese superoxide dismutase expression (Mn-SOD) and that of glutathione content (GSH) as well as by regulating the decrease of glutathione synthetase and/or by increasing oxidant levels. In addition, Tat induces the production of free radicals in several types of cells susceptible to induce carcinogenesis [26]. Following this inflammatory/oxidizing environment, a marker of lipid peroxidation increases and stimulates the body to produce glutathione for the regulation and maintenance of redox balance.

We noted a significant increase in MDA concentrations in patients taking their ARVs irregularly, *p < 0.0001*. This is due to the fact that lipid peroxidation is more pronounced in the absence of ARV treatment [27]. The correlation between the different immunological and oxidative parameters in patients with IL-6 concentrations ≥ 37 showed a weak positive correlation (r = 0.3, *p < 0.05*) between IL-6 and MDA, a strong negative correlation between FRAP and MDA (r = -0.5, *p < 0.01*) as well as a strong positive correlation between MDA and GSH (r = 0.6, *p < 0.01*). In patients with IL-6 concentrations < 37 pg/mL, there was a strong positive correlation between MDA and GSH (r = 0.6, *p < 0.01*). The work of Teto et al., on lipid peroxidation in HIV-positive patients in 2013 in Cameroun showed a reduction in total antioxidant capacity and an increase in their blood GSH content [27]. Ligia et al., (2016) in the United States showed that these changes are more pronounced in patients who have never been treated than in patients on ARVs, since ARVs restore the number of CD4 + T cells, but at the same time assesses the equilibrium of the oxidation state [28].

## Conclusion

Investigating the relation between Interleukin-6 concentration and oxidative status of HIV-infected patients on ARV treatment with or at risk of Kaposi’s disease, the frequency of the HHV-8 antigen was 57.5% and the prevalence of clinical signs of Kaposi’s disease was 3.4%. There was a significant increase in IL-6 concentrations in patients positive for the HHV-8 antigen for IL-6 concentrations < 37 pg/mL. Concentrations of MDA and GSH increased significantly with HHV-8 infection while total antioxidant capacity (FRAP) concentrations decreased significantly with HHV-8 infection. Finally, this paper suggests that HIV-infected patients with HHV-8 infection in association with inflammation and oxidative stress could expose the patient to Kaposi disease.

## Data Availability

All data generated or analyzed during this study are included in this published article.

## Abbreviations

AIDS: Acquired Immuno-deficiency Syndrom
ARV: Anti-retrovirals
DNA: Deoxyribonucleic Acid
ELISA: Enzyme- Linked Immunosorbent Assay
FRAP: Ferric Reduicing Antioxidant Power
GSH: Glutathion Reduced
HHV-8: Human Herpes Virus type 8
HIV: Human Immuno-deficiency Virus
IL: Interleukin
KD: Kaposi Disease
KSHV: Kaposi’s Sarcoma Herpes Virus
MDA: Malondialdehyde
MIP: Macrophage Inflammatory Protein
PLHIV: People living with HIV
ROS: Reactive Oxygen Species
